# Mass-Fix better predicts for PFS and OS than standard methods among multiple myeloma patients participating on the STAMINA trial (BMT CTN 0702 /07LT)

**DOI:** 10.1038/s41408-022-00624-6

**Published:** 2022-02-10

**Authors:** Angela Dispenzieri, Amrita Krishnan, Bonnie Arendt, Beth Blackwell, Paul K. Wallace, Surendra Dasari, Dan T. Vogl, Yvonne Efebera, Mingwei Fei, Nancy Geller, Sergio Giralt, Theresa Hahn, Alan Howard, Mindy Kohlhagen, Heather Landau, Parameswaran Hari, Marcelo C. Pasquini, Muzaffar H. Qazilbash, Philip McCarthy, Nina Shah, David H. Vesole, Edward Stadtmauer, David Murray

**Affiliations:** 1 Division of Hematology, Mayo Clinic, Rochester, MN USA; 2 Department of Laboratory Medicine, Mayo Clinic, Rochester, MN USA; 3 City of Hope, Los Angeles, CA USA; 4 The Emmes Company, Rockville, MD USA; 5 Roswell Park Cancer Institute, Buffalo, NY USA; 6 Abramson Cancer Center, University of Pennsylvania, Philadelphia, USA; 7 Ohio State University, Columbus, OH USA; 8 Medical College of Wisconsin, Milwaukee, WI USA; 9 National Heart, Lung, and Blood Institute, Rockville, MD USA; 10 Memorial Sloan Kettering Cancer Center, New York, NY USA; 11 Center for International Blood and Marrow Transplant Research, Minneapolis, MN USA; 12 The University of Texas MD Anderson Cancer Center, Houston, TX USA; 13 University of California San Francisco, San Francisco, USA; 14 Hackensack University, Hackensack, NJ USA

**Keywords:** Myeloma, Myeloma

## Abstract

Measuring response among patients with multiple myeloma is essential for the care of patients. Deeper responses are associated with better progression free survival (PFS) and overall survival (OS). To test the hypothesis that Mass-Fix, a mass spectrometry-based means to detect monoclonal proteins, is superior to existing methodologies to predict for survival outcomes, samples from the STAMINA trial (NCT01109004), a trial comparing three transplant approaches, were employed. Samples from 575 patients from as many as three time points (post-induction [post-I; pre-maintenance [pre-M]; 1 year post enrollment [1YR]) were tested when available. Four response parameters were assessed: Mass-Fix, serum immunofixation, complete response, and measurable residual disease (MRD) by next generation flow cytometry. Of the four response measures, only MRD and Mass-Fix predicted for PFS and OS at multiple testing points on multivariate analyses. Although MRD drove Mass-Fix from the model for PFS at post-I and pre-M, 1YR Mass-Fix was independent of 1YR MRD. For OS, the only prognostic pre-I measure was Mass-Fix, and the only 1YR measures that were prognostic on multivariate analysis were 1YR MRD and 1YR Mass-Fix. SIFE and CR were not. Mass-Fix is a powerful means to track response.

## Introduction

Measuring response among patients with multiple myeloma (MM) is essential for the care of patients [1]. Deeper responses have been associated with better progression free survival (PFS) and overall survival (OS) [2]. Serum (SIFE) and urine immunofixation are the first steps to documenting complete response; thereafter, the bone marrow is tested for the existence of plasma cells by morphology, and next generation flow and/or sequencing are used to document measurable residual disease status. Mass spectrometry of blood by (Mass-Fix) confers better specificity and sensitivity than SIFE [3–7]. The benefit of the increased analytical sensitivity was seen in screening patients for plasma cell disorders, but no published prospective studies have documented benefits for treatment response. There are emerging data that mass spectrometric measurements of blood may be superior to conventional measures for both myeloma and AL amyloidosis [3, 5, 8, 9]. Given that Mass-Fix testing is performed on serum as opposed to bone marrow, there are economical and patient care benefits inherent in the assay. To test the hypothesis that Mass-Fix is superior to existing methodologies, samples from the Blood and Marrow Transplant Clinical Trials Network 0702 and 07LT (STAMINA trial)), a trial comparing 3 transplant approaches among patients who have already received a variety of induction regimens, were utilized [10]. The primary endpoint of this correlative study was to determine if serum Mass-Fix was prognostic for PFS and OS. A secondary endpoint was to determine the utility of Mass-Fix to predict for measurable residual disease (MRD) status.

## Methods

### Data sources

This study was an ancillary study to the STAMINA Trial, which include the parent BMT CTN 0702 (NCT 01109004) and the follow on trial BMT CTN 07LT (NCT02322320). The Center for International for Blood and Marrow Transplant Research (CIBMTR) outcomes database was used to supplement data from both clinical trials. Lastly, the data on MRD was obtained through a STAMINA trial ancillary study Prognostic Immunophenotyping for Myeloma Response (PRIMeR). Patients with available samples who enrolled in the STAMINA trial were eligible for this study. Consent for enrollment of all study subjects was managed by local IRBs, the CTN, and the CIBMTR. The Mayo Clinic IRB approved the study protocol for this correlative project.

### Samples and laboratory assays

Samples from 614 of 758 patients enrolled on the trial were obtained, and three time points (enrollment post-induction (post-I); pre-maintenance (pre-M); 1-year post enrollment (1YR)) were tested when available. The patient population tested at each time point was comprised of patients who were without progression and had available disease burden assessment and samples for Mass fix. SPEP and Mass-Fix were performed as previously described [7]. Median (range) time from the post-I enrollment sample to pre-M was 172 (56–405) days and to 1YR was 371 (277–565) days. The pre-M sample had the most variability in time to begin maintenance, since this depended on the treatment arm: Auto/Maintenance: 98 days (range 62–207); Auto/ RVD/maintenance: 209 days (range 56–405); and Auto/Auto/Maintenance: 190 days (range 62–384), *p* < 0.0001.

Spectra were evaluated by BA and DM in a blinded fashion. To avoid assignment of a post-treatment oligoclonal band as the patients’ clone, baseline samples (the first Mass-Fix measurements occurred after patients had completed induction) were included in the study if the baseline Mass-Fix result: (1) was negative; (2) matched CTN reported “at diagnosis” isotype; or (3) did not match the CTN “at diagnosis” isotype, but was concordant with the reported FLC diagnosis free light chain or was found at repeated MASS-FIX time points. This methodology excluded 39 patients leaving 575 patients for this study (Supplementary Fig. 1). According to protocol, high-risk MM was defined as beta-2 microglobulin >5 mg/ L or presence of t(4;14), t(14:16), t(14;20), deletion 17p, aneuploidy by FISH or metaphase cytogenetics or deletion 13q by metaphase cytogenetics.

MRD was determined by multiparametric flow cytometry (MFC) of the bone marrow aspirate samples as part of the optional PRIMeR study. It was recommended that all patients have an enrollment/post-I MRD sample collected and a pre-M MRD sample collected. At other time points, bone marrow biopsy was required only to confirm a complete response. The MFC methodology has a minimum detection sensitivity of 10^−5^ [11, 12]. Aliquots of 2 mL of marrow were collected in one sodium heparin and shipped at room temperature priority overnight from individual centers to the central Flow and Image Cytometry Laboratory at Roswell Park Comprehensive Cancer Center to perform all MFC analyses. Samples that were older than 48 h or with viability <85% were not processed. Upon arrival at the central flow laboratory, an automated WBC and lymphocyte count was performed using a standard stain/lyse/wash/fix procedure for routine flow cytometric analysis. Bone marrow aspirates were washed once with FCM Buffer (containing 0.5% BSA, 0.1% Na azide, and 0.004% disodium EDTA in PBS pH 7.2), resuspended to their original volume and incubated for 10 min with normal mouse IgG (10 μg/test) to block Fc receptors. Cells were then aliquoted into 10 × 75 mm tubes (200 µL per tube) and incubated for 20 min with mAbs. All three tubes had the following backbone: CD38 V450 (HB7), Live Dead Aqua (Thermo Fisher), CD45 FITC (2D1), CD138 PerCPCy5.5 (MI15). Tube 1 also contained: CD56 PE (NKH-1: Beckman Coulter), CD19 PECy7 (J3-119: Beckman Coulter), CD20 APC (L27). Tube 2 also contained: cLambda light chain PE (Dako), CD19 PECy7, cKappa light chain APC (Dako). Tube 3 contained: CD117 PE (104D2: Beckman Coulter), CD27 PECy7 (1A4CD27: Beckman Coulter), CD28 APC (CD28.2). Unless otherwise indicated mAbs were from BD Bioscience. Next red blood cells were lysed with BD FACSLyse, and the resulting cell pellets were washed once with FCM buffer before fixing Tube 1 and 3 in 0.5% methanol free formaldehyde (MFF; Polysciences, Warrington, PA). For intracellular light chain staining, Tube 2 was fixed with 2% MFF for 10 min, washed, permeabilized for 10 min with Caltag B buffer, then washed, and finally fixed with 0.5% MFF.

Cytofluorometric analysis was performed typically within 24 h after staining using a BD FACSCanto flow cytometer with DiVa software, quality controlled daily with CS&T software. Data on the flow cytometer were collected using a forward scatter threshold to eliminate cellular debris for up to 3 min for a minimum of 2.5 × 10^5^ events and a target of 1.5 × 10^6^ events. Within the first year of the study the minimum goal was increased to 1 × 10^6^ events for a sensitivity of 0.001%. Data were analyzed using a variable, mononuclear cell gate based on forward and side scatter. A qualitative assessment of MRD negative, positive or equivocal was determined based on a quantitative analysis of the three 6-color tubes detailed above. After the study was completed, all immunophenotyping reports were reviewed for consistency. Data were analyzed with WinList (Verity Software House, Topsham, ME) using sequential gates to eliminate doublets, debris, aggregates and defining plasma cells using CD45, CD38, and CD138.

### Statistical analysis

For the purpose of statistical analyses, an MRD equivocal result was categorized as MRD positive. OS and PFS were analyzed by the Kaplan–Meier method and differences between curves were tested for significance using Cox proportional hazards at each time point, post-I, pre-M and at 1YR. Follow-up information was based on BMT CTN 0702 and on the subsequent follow-up study, BMT CTN 07LT to derive long-term follow-up [10, 13]. PFS was measured from enrollment to progression or death from any cause. OS was measured from enrollment to death. Multivariate Cox proportional hazards models using stepwise regression were developed to explore the independent effect of the different response measures on PFS and OS and were performed independently at each time point. Two models were assessed for each time point, one considering interactions of disease assessment and MRD with treatment arm and age and a second model forcing MM risk status. Variables were retained in the model for levels of significance of *p* < 0.05 Analyses were performed using JMP 14, SAS NC.

## Results

### Patient characteristics

The on-study post-I patient characteristics are shown in Table 1 for those patients who had adequate samples to participate in this correlative lab study. Median age was 57 years, and 62% were male. At post-I 17% were reported to be in CR or stringent CR, 29% in VGPR or nCR, 44% in PR, and the remaining 9% with stable, progressive or not evaluated disease.

**Table 1:** Patient characteristics (*n* = 575).

Characteristics	*N* (%)
Age	57 (20, 71)
Male	335 (62)
High risk^a^	201 (35)
*Induction*	
Lines, med (range)	1 (1, 3)
Triplets	427 (96)
Doublets	15 (3)
Missing	3 (1)
*Treatment arm*	
Auto-maintenance	198 (34)
Auto-RVD	195 (34)
Auto-Auto	182 (32)
*Reported diagnostic isotype*, *n* (%)	
GK/GL	224 (39)/103 (18)
AK/AL	45 (8)/39 (7)
Free K/free L	75 (13)/31 (5)
MK/ML	3 (<1)/0 (0)
DK/DL	1 (<1)/3 (<1)
≥Biclonal	15 (2)
Neg	1 (<1)
Missing	35 (6)
*CTN response at entry*	
≥CR	96 (18)
VGPR^b^	169 (29)
PR	255 (44)
<PR or not evaluated	55 (9)
Positive Mass-Fix at enrollment	437 (76)

^a^High risk was defined as beta-2 microglobulin >5 mg/L or presence of t(4;14), t(14:16), t(14;20), deletion 17p, aneuploidy by FISH or metaphase cytogenetics or deletion 13q by metaphase cytogenetics.

^b^Includes nCR and VGPR.

#### Response and comparison of response variables

At the 3 time points, the rates of CR (and ≥VGPR) were as follows: Post-I, 17% (and 46%); Pre-M, 37% (and 73%); and 1YR 47% (and 84%). The rates of negative Mass-Fix among those patients achieving VGPR or better at Post-I, Pre-M, and 1YR were 42%, 41%, and 58%, respectively. The respective rates of negative SIFE for these same 3 measurement points were 59%, 62%, and 66%. Rates of MRD negativity at the three time points, among those patients achieving greater than VGPR, were: Post-I, 68%, Pre-M, 87%, and 1YR, 92%.

**Fig. 1:**
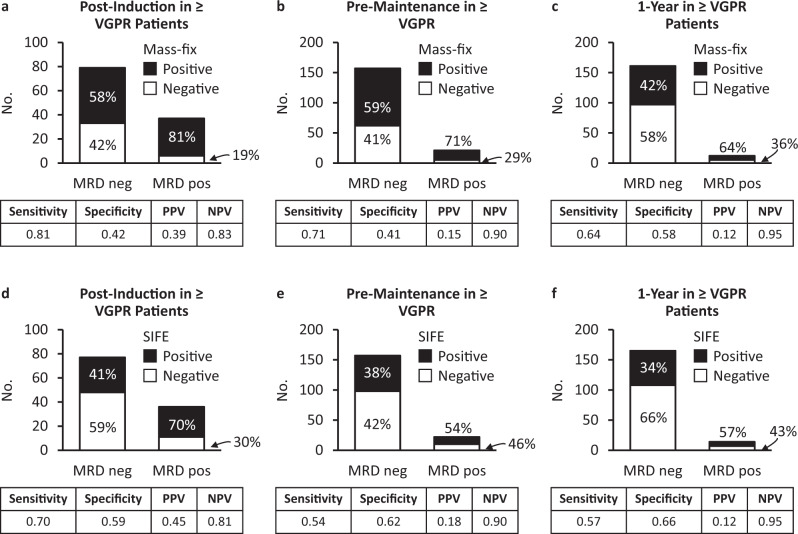
Performance of serum Mass-Fix as compared to bone marrow MRD. **a**–**c** performance of Mass-Fix among patients in CR or better at 3 time points; **d**–**f** performance of SIFE among patients in VGPR or better at 3 time points.

The relationships between MRD and SIFE/Mass-Fix among patients with VGPR or better are shown in Fig. 1. For these analyses, the assumption made was that NGF MRD was the gold standard. With that assumption, the negative predictive value (NPV) of both Mass-Fix and SIFE appeared comparable, with better sensitivity of Mass-Fix but poor specificity and positive predictive value (PPV) for both Mass-Fix and SIFE. Analyses were limited by the fact that most patients did not have MRD testing; frequencies of testing and individual results across tests for all patients are shown in Supplementary Figs. 2 and 3.

### Comparative utility of Mass-Fix to predict for PFS

**Fig. 2:**
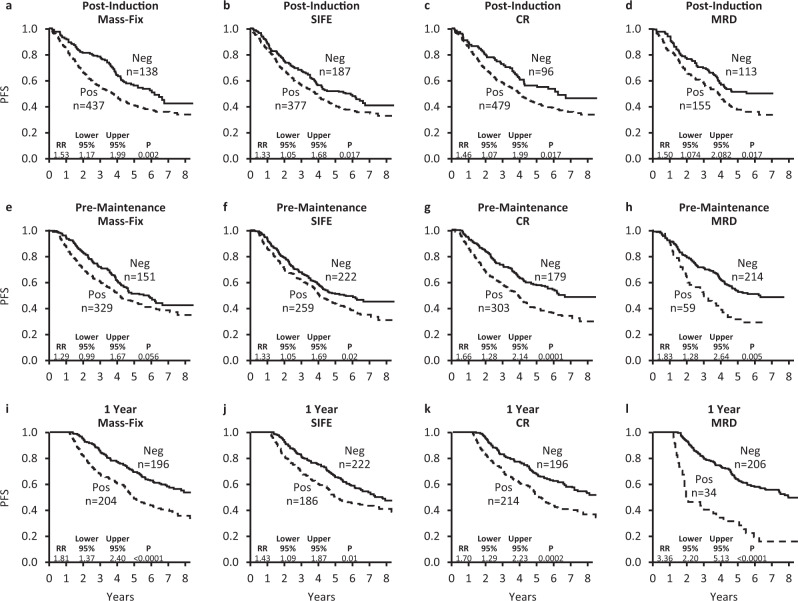
Progression free survival based on response measurement at the time points. **a**–**d** post-induction sample; **d**–**h** pre-maintenance sample; **i**–**l** 1 year post enrollment sample.

There have been 330 progression events and 341 progression or death events among the 575 patients. The median follow-up of the non-progressors is 6.1 years. Six-year PFS for the correlative population was 41%. Each of the post-I response measures (Mass-Fix, SIFE, MRD, and CR) predicted for PFS with hazard ratios ranging from 1.3 to 1.5 (Table 2 and Fig. 2a–d); however, on multivariate, MRD bone marrow status drove the other 3 response measures from the model. Upon the addition of MM risk to the model, only it and MRD status were significant predictors for PFS using the post-I time point samples. CR, SIFE and Mass-Fix were not prognostic, presumably due to the serum half-lives of immunoglobulins at this early time point. Treatment arm and age also were not prognostic.

**Table 2:** PFS univariate and multivariate.

	n/N	Univariate			Multivariate 1			Multivariate 2		
		HR	95%CI	*p* value	HR	95%CI	*p* value	HR	95%CI	*p* value
*Post-induction*										
Myeloma high-risk	201/574	1.45	1.17, 181	0.0009	NI	NI	NI	1.62	1.16, 2.28	0.0005
MRD positive	155/268	1.50	1.07, 2.08	0.017	1.5	1.07, 2.08	0.017	1.63	1.16, 2.27	0.0005
Mass-Fix positive	437/575	1.53	1.17, 1.99	0.002	–	–	NS	–	–	NS
<CR	479/575	1.46	1.07, 1.99	0.017	–	–	NS	–	–	NS
SIFE positive	387/574	1.33	1.05, 1.68	0.017	–	–	NS	–	–	NS
*Pre-maintenance*										
Myeloma high-risk (post-I)	*See above*	1.45	1.17, 181	0.0009	NI	NI	NI	1.73	1.24, 2.41	0.001
MRD positive	59/273	1.83	1.28, 2.64	0.005	1.83	1.28, 2.64	0.005	1.93	1.34, 2.78	0.0004
Mass-Fix positive	329/480	1.29	0.99, 1.67	0.056	–	–	NS	–	–	NS
<CR	303/482	1.66	1.28, 2.14	0.0001	–	–	NS	–	–	NS
SIFE positive	259/481	1.33	1.05, 1.68	0.022	–	–	NS	–	–	NS
*1-year (landmark)*										
Myeloma high-risk (post-I)	*See above*	1.45	1.17, 181	0.0009	NI	NI	NI	1.91	1.31, 2.78	0.0008
MRD positive	42/251	3.36	2.20, 5.13	<0.0001	3.01	1.93, 4.70	<0.0001	3.24	2.07, 5.06	<0.0001
Mass-Fix positive	221/423	1.81	1.37, 2.40	<0.0001	1.62	1.11, 2.35	0.012	1.67	1.14, 2.42	0.007
<CR	232/434	1.70	1.29, 2.23	<0.0001	–	–	NS	–	–	NS
SIFE positive	203/432	1.43	1.09, 1.87	0.01	–	–	NS	–	–	NS

Not shown, but treatment arm and age were not significant predictors for PFS.

*HR* hazard ratio, *NI* not included, *NS* not significant.

As shown in Table 2 and Fig. 2e–h, on univariate analysis for PFS using pre-*M* values, the relative risk of progression ranged from 1.3 to 1.8. MRD forced the other 3 response variables out of the model in multivariate analysis. MRD positivity pre-*M* retained its predictive value even when baseline MM Risk was added.

**Fig. 3:**
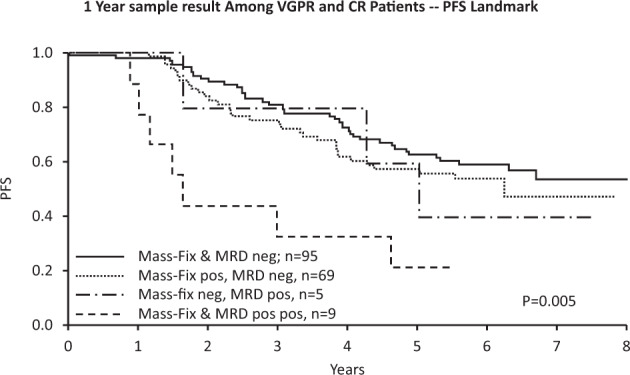
Interaction between Mass-Fix and MRD status and PFS using 1-year post enrollment MRD and Mass-Fix results.

At the 1YR time point (Table 2 and Fig. 2i–l), 79 patients had progressed, so 1YR measures were analyzed as 1-year landmark analyses. On univariate analysis, each of the 1YR variables were predictive for PFS with risk ratios ranging from 1.4 to 3.9; however, in multivariate analysis, only 1YR Mass-Fix, 1YR MRD status and baseline MM risk were prognostic. Treatment arm, age, 1YR CR adjudication, and 1YR SIFE were not prognostic in multivariate analysis. Figure 3 illustrates the additive value of 1YR Mass-Fix and 1YR MRD.

### Comparative utility of Mass-Fix to predict for OS

**Fig. 4:**
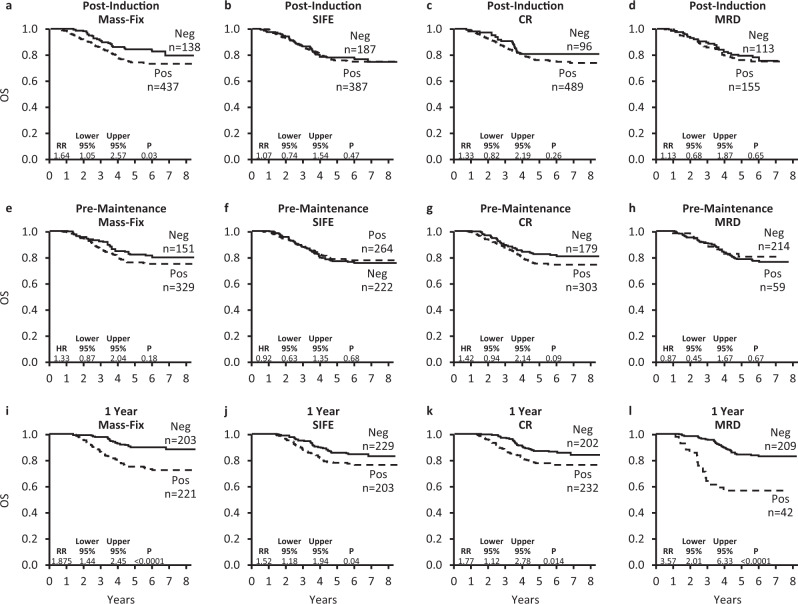
Overall survival based on response measurement at specific time points. **a**–**d** Post-induction sample; **d**–**h** pre-maintenance sample; and **i**–**l** 1 year post enrollment sample.

With a median follow-up of 6 years, there have been 136 deaths, and 6-year OS was 76%. Table 3 and Fig. 4 demonstrate OS outcomes based on MM risk as well as the four responses measurements at the three different times points. The only post-I response variable that predicted for death was Mass-Fix with a RR of death of 1.64 (1.05, 2.57, *p* = 0.03). Post-I CR, SIFE, and MRD did not predict for OS. At the pre-M time point none of these four response variables were predictive for OS. Response measures at the 1YR mark were also evaluated, and risk ratios for death ranged between 1.5 and 3.6 with 1YR MRD status having the greatest impact. On multivariate analysis, predictors for OS were 1YR Mass-Fix, MM risk, and 1YR MRD with relative RR of death of 2.0, 2.3, and 2.8, respectively. It should be noted that of the 434 patients assessed at 1YR, only 251 (58%) had MRD testing (Supplementary Figs. 2 and 3).

**Table 3:** Overall survival, univariate and multivariate analyses.

Baseline	n/N	Univariate for OS			Multivariate 1			Multivariate 2		
		Baseline								
		HR	95%CI	*p* value	HR	95%CI	*p* value	HR	95%CI	*p* value
Myeloma high-risk	201/574	1.76	1.26, 2.46	0.001	NI	NI	NI	1.83	1.30, 2.56	0.0005
MRD positive	155/268	1.13	0.68, 1.87	0.65	–	–	–	–	–	–
Mass-Fix positive	437/575	1.64	1.05, 2.57	0.03	1.64	1.05, 2.57	0.03	1.74	1.11, 2.74	0.0151
<CR	479/575	1.33	0.82, 2.19	0.26	–	–	–	–	–	–
SIFE pos	387/574	1.07	0.74, 1.54	0.47	–	–	–	–	–	–
										NS
*Pre-maintenance*										
Myeloma high-risk (post-I)	*See above*	1.76	1.26, 2.46	0.001	NI	NI	NI	1.76	1.26, 2.46	0.001
MRD positive	59/273	0.87	0.45, 1.67	0.67	–	–	–	NS	NS	NS
Mass-fix positive	329/480	1.33	0.87, 2.04	0.18	–	–	–	NS	NS	NS
<CR	303/482	1.42	0.94, 2.14	0.09	–	–	–	NS	NS	NS
SIFE pos	259/481	0.92	0.63, 1.35	0.68	–	–	–	NS	NS	NS
*1-year*										
Myeloma high-risk (post-I)	*See above*	1.76	1.26, 2.46	0.001	NI	NI	NI	2.29	1.31, 4.02	0.004
MRD positive	42/251	3.57	2.01, 6.33	<0.0001	2.77	1.50, 5.12	0.0012	2.83	1.53, 5.24	0.0009
Mass-Fix positive	221/423	1.88	1.44, 2.45	<0.0001	1.93	1.04, 3.56	0.036	1.96	1.06, 3.61	0.03
<CR	232/434	1.77	1.12, 2.78	0.014	NS	NS	NS	NS	NS	NS
SIFE pos	203/432	1.52	1.18, 1.96	0.0014	NS	NS	NS	NS	NS	NS

Not shown, but treatment arm and age were not significant factors.

*HR* hazard ratio, *NI* not included, *NS* not significant.

## Discussion

Herein we have demonstrated that serum Mass-Fix consistently outperformed CR and SIFE as response indicators for survival measures. The primary endpoint of our study was met in that Mass-Fix was prognostic for both PFS and OS on univariate and multivariate at most time points. At post-I and pre-M, none of the response criteria that relied on clearance of immunoglobulin from the circulation could compete with NGF MRD assessment to predict for PFS, possibly due to long half-lives of monoclonal proteins as a consequence of immunoglobulin recycling [14], the wide range of time that the pre-M time point encompassed due to trial design, post-transplant/post consolidation oligoclonal banding, and, perhaps, missing data/data quality in this multicenter cooperative study. In terms of the 1YR response measurements, however, Mass-Fix was the only response parameter that was independent of MRD for PFS prognostication on multivariate analysis. Surprisingly, at post-I measurement, Mass-Fix was the response measure that predicted for OS. Had more than 47% of patients had MRD testing at post-I, perhaps that measure would have also been significant at that time point. For the 1YR measures, Mass-Fix and MRD were the two response variables to predict for OS. None of the blood-based measurements were prognostic at the Pre-M time point presumably due to reasons mentioned above.

This study adds to the growing body of literature demonstrating how valuable the mass spectrometry of serum can be to detect residual disease when SIFE or even bone marrow studies do not [5, 8, 9, 15–18]. Although all of these studies impart the same message—that mass spectrometry of blood is very sensitive and at times even more sensitive than bone marrow—they are small, have limited time points, and/or limited follow-up.

Not surprisingly, there were discrepant results between measures of response; the PETHMA group has illustrated the same [15]. Comparing blood (and or urine) to bone marrow results is inherently challenging given disparate kinetics of disappearance of myeloma cells versus intact immunoglobulins [14]. Discrepancies can also arise from the patchiness of plasma cell involvement in intramedullary and extramedullary spaces. The incorporation of advanced imaging in myeloma response criteria speaks to this second concern [1].

The NPV of a negative Mass-Fix predicting for an MRD negative marrow improved over time, which was consistent with a deepening and time-dependent response. SIFE appeared to perform nearly as well as Mass-Fix in terms of NPV; however, the multivariate analyses demonstrated superior prognostic power for Mass-Fix’s ability to predict for both PFS and OS. The fact that the sensitivity of Mass-Fix to predict for an MRD negative bone marrow appeared to decrease at successive time points is at first glance puzzling; however, this can be explained by the fact that Mass-Fix and MRD by NGF are independently prognostic—i.e. complementary to each other—for both PFS and OS at the 1YR measurement. Moreover, analyses are compromised by relatively low numbers patients with MRD testing.

It is remarkable that a single blood test was able to out-perform a composite endpoint of adjudicated CR to predict for PFS and OS. This finding is likely due to issues of sensitivity and specificity of IFE, which is integral to the definition of CR. Mass-Fix has greater sensitivity than IFE [3, 6, 7], but importantly, greater specificity which is further enhanced by having a baseline sample. In a study of 226 patients from the Olmsted monoclonal gammopathy of undetermined significance screening cohort who were initially negative for monoclonal gammopathy by SPEP but subsequently developed a monoclonal gammopathy during the follow up period, the M-proteins were detectable in the original screening sample in 11% and 50% of patients by IFE and Mass-Fix, respectively [4].

There are limitations to this study. First, despite the fact that this was a prospective trial, there was incomplete testing for the cohort (only about 50% of CR and VGPR patients had MRD testing) though there was no obvious systematic reason for limited research samples for Mass-Fix and MRD testing. Second, patients were not recipients of therapeutic monoclonal antibodies, making this cohort less reflective of a contemporary cohort. Routine Mass-Fix can distinguish therapeutic monoclonal antibodies, which can confound response assessments using standard SIFE techniques [19, 20]. Third, there was no “at diagnosis” sample to definitively determine any given patient’s light chain mass, making it possible that a small post-induction oligoclonal band could have been assigned as a patient’s monoclonal protein to follow throughout the study. At the first Mass-Fix measure, 17% of patients were already in CR and another 30% were in VGPR, potentially underutilizing the added sensitivity and specificity that comes with having a known light chain mass for a given patient. Fourth, there was no central SIFE testing done whereas the Mass-Fix was centrally run. Fifth, follow-up is limited to 6 years, which is short to detect survival differences. Each of these limitations likely contributes to underestimating the full utility of Mass-Fix, and through longer follow-up and additional studies, the full value of Mass-Fix will be better elucidated.

### Reporting summary

Further information on research design is available in the Nature Research Reporting Summary linked to this article.

## Supplementary information

Reporting Summary

Supplementary Figure

## References

[1] Kumar S, Paiva B, Anderson KC, Durie B, Landgren O, Moreau P (2016). International Myeloma Working Group consensus criteria for response and minimal residual disease assessment in multiple myeloma. Lancet Oncol.

[2] Avet-Loiseau H, Ludwig H, Landgren O, Paiva B, Morris C, Yang H (2020). Minimal residual disease status as a surrogate endpoint for progression-free survival in newly diagnosed multiple myeloma studies: a meta-analysis. Clin Lymphoma Myeloma Leuk.

[3] Dispenzieri A, Arendt B, Dasari S, Kohlhagen M, Kourelis T, Kumar SK (2020). Blood mass spectrometry detects residual disease better than standard techniques in light-chain amyloidosis. Blood Cancer J.

[4] Murray D, Kumar SK, Kyle RA, Dispenzieri A, Dasari S, Larson DR (2019). Detection and prevalence of monoclonal gammopathy of undetermined significance: a study utilizing mass spectrometry-based monoclonal immunoglobulin rapid accurate mass measurement. Blood Cancer J.

[5] Mills JR, Barnidge DR, Dispenzieri A, Murray DL (2017). High sensitivity blood-based M-protein detection in sCR patients with multiple myeloma. Blood Cancer J.

[6] Milani P, Murray DL, Barnidge DR, Kohlhagen MC, Mills JR, Merlini G (2017). The utility of MASS-FIX to detect and monitor monoclonal proteins in the clinic. Am J Hematol.

[7] Mills JR, Kohlhagen MC, Dasari S, Vanderboom PM, Kyle RA, Katzmann JA (2016). Comprehensive assessment of M-proteins using nanobody enrichment coupled to MALDI-TOF mass spectrometry. Clin Chem.

[8] Derman BA, Stefka AT, Jiang K, McIver A, Kubicki T, Jasielec JK (2021). Measurable residual disease assessed by mass spectrometry in peripheral blood in multiple myeloma in a phase II trial of carfilzomib, lenalidomide, dexamethasone and autologous stem cell transplantation. Blood Cancer J.

[9] Puíg N, Contreras T, Paiva B, Cedena MT, Martinez-Lopez J, Oriol A (2020). Analysis of treatment efficacy in the GEM-CESAR trial for high-risk smoldering multiple myeloma patients: Comparison between the standard and IMWG MRD criteria and QIP-MS including FLC (QIP-FLC-MS). J Clin Oncol.

[10] Stadtmauer EA, Pasquini MC, Blackwell B, Hari P, Bashey A, Devine S (2019). Autologous transplantation, consolidation, and maintenance therapy in multiple myeloma: results of the BMT CTN 0702 Trial. J Clin Oncol.

[11] Soh KT, Wallace PK. Monitoring of measurable residual disease in multiple myeloma by multiparametric flow cytometry. Curr Protoc Cytom. 2019;90:e63. 10.1002/cpcy.63 PMC6788635 31608132

[12] Tario JD, Wallace PK. Reagents and cell staining for immunophenotyping by flow cytometry. In: McManus LM, Mitchell RN, editors. Pathobiology of human disease. 1 ed. Elsevier, Academic Press; 2014. p. 3678–701. 10.1016/B978-0-12-386456-7.07104-5.

[13] Hari P, Pasquini MC, Stadtmauer EA, Fraser R, Fei M, Devine SM (2020). Long-term follow-up of BMT CTN 0702 (STaMINA) of postautologous hematopoietic cell transplantation (autoHCT) strategies in the upfront treatment of multiple myeloma (MM). J Clin Oncol.

[14] Junghans RP, Anderson CL (1996). The protection receptor for IgG catabolism is the beta2-microglobulin-containing neonatal intestinal transport receptor. Proc Natl Acad Sci USA.

[15] Paiva B, Martinez-Lopez J, Vidriales MB, Mateos MV, Montalban MA, Fernandez-Redondo E (2011). Comparison of immunofixation, serum free light chain, and immunophenotyping for response evaluation and prognostication in multiple myeloma. J Clin Oncol.

[16] Abeykoon JP, Murray DL, Murray I, Jevremovic D, Otteson GE, Dispenzieri A (2021). Implications of detecting serum monoclonal protein by MASS-fix following stem cell transplantation in multiple myeloma. Br J Haematol.

[17] Santockyte R, Jin C, Pratt J, Ammar R, Desai K, Bolisetty M (2021). Sensitive multiple myeloma disease monitoring by mass spectrometry. Blood Cancer J.

[18] Foureau D, Bhutani M, Guo F, Rigby K, Leonidas M, Tjaden E (2021). Comparison of mass spectrometry and flow cytometry in measuring minimal residual disease in multiple myeloma. Cancer Med.

[19] Mills JR, Kohlhagen MC, Willrich MAV, Kourelis T, Dispenzieri A, Murray DL (2018). A universal solution for eliminating false positives in myeloma due to therapeutic monoclonal antibody interference. Blood.

[20] Moore LM, Cho S, Thoren KL (2019). MALDI-TOF mass spectrometry distinguishes daratumumab from M-proteins. Clin Chim Acta.

