# Supplementary material for: Mass-Fix better predicts for PFS and OS than standard methods among multiple myeloma patients participating on the STAMINA trial (BMT CTN 0702 /07LT)
Source: Blood Cancer J. 2022 Feb 10;12(2):27. doi: 10.1038/s41408-022-00624-6 (PMC8831597; doi:10.1038/s41408-022-00624-6)
Supplement: Supplementary file 2 — Supplementary Figure [file 41408_2022_624_MOESM2_ESM.docx]

Randomly assigned N=758

# ASCT +ASCT +Len arm

Pts=247; Samples=182

### ≥ VGPR = 88

MRD testing=83

# ASCT +RVD +Len arm

Pts=254; Samples =195

### ≥ VGPR = 93

MRD testing=88

# ASCT +Len arm

Pts=257; Samples=198

### ≥ VGPR = 85

MRD testing=97

## *Started maintenance*

Pts=206; Samples=146

### ≥ VGPR = 107

MRD testing=79

## *Started maintenance*

Pts=212; Samples=162

### ≥ VGPR = 127

MRD testing=97

## *Started maintenance*

Pts=242; Samples=172

### ≥ VGPR = 117

MRD testing=97

## *1 year sample*

Samples=182

### ≥ VGPR = 102

MRD testing=71

## *1 year sample*

Samples=195

### ≥ VGPR = 125

MRD testing=81

## *1 year sample*

Samples=198

### ≥ VGPR = 114

MRD testing=99

Legend: Negative Positive Not done

# Post-Induction

Response Mass-Fix SIFE MRD


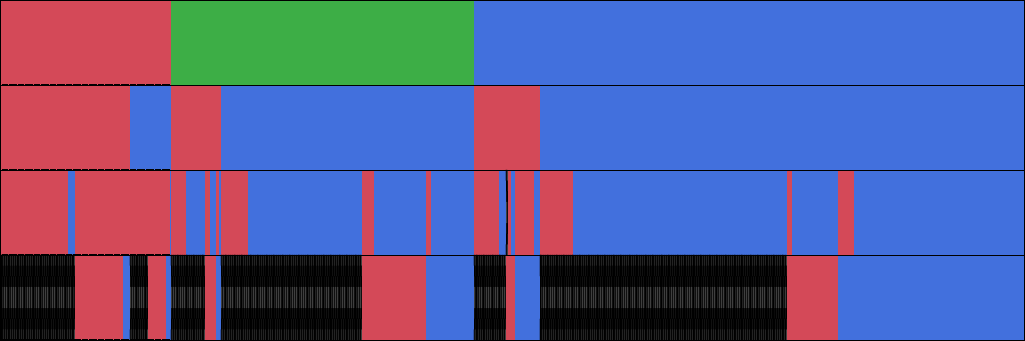


**CR, 17%**

**VGPR, 29%**

**<VGPR**

# Pre-Maintenance

Response Mass-Fix SIFE MRD


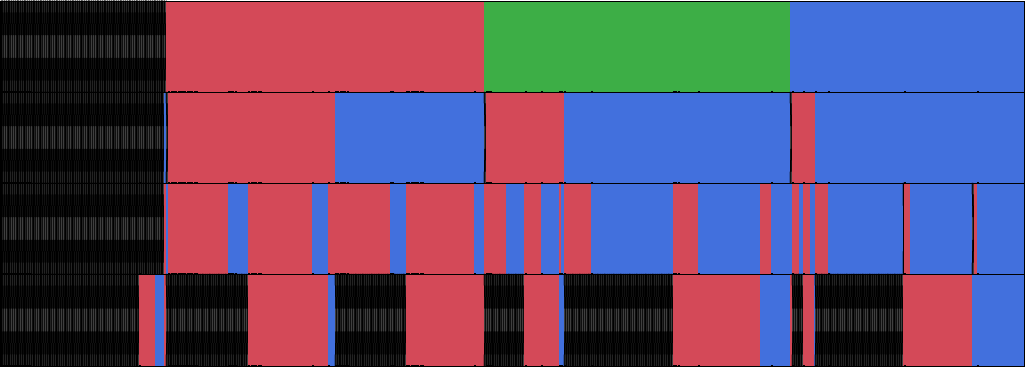


**No assignment**

**CR, 37%**

**VGPR, 36%**

**<VGPR**

# 1YR post enrollment

Response Mass-Fix SIFE MRD


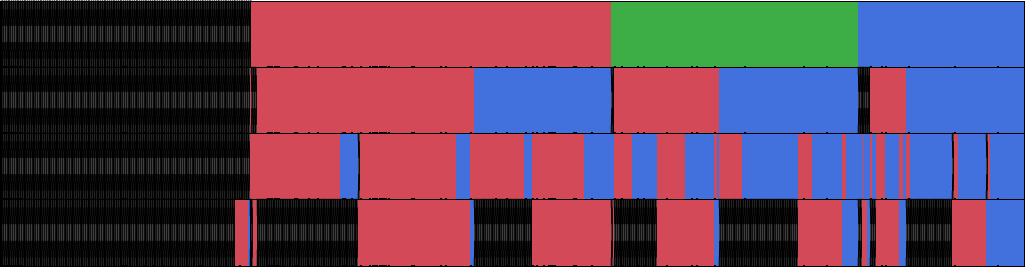


**No assignment**

**CR, 47%**

**VGPR, 37%**

**<VGPR**

Each line (column) represents a separate participant (n=575) Hematologic response rates are calculated as % assessed.

Legend: Negative (or CR) Positive (or not CR) Not done

- 1.
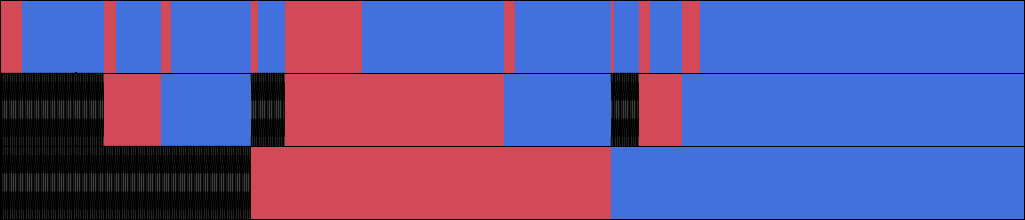
Complete response Post-I

Pre-M 1YR

- 1.
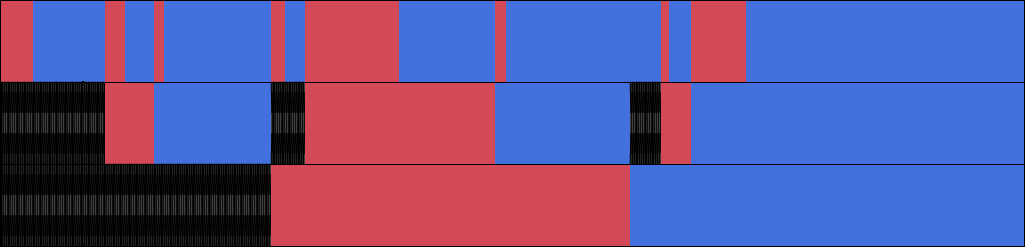
Mass-Fix Post-I

Pre-M 1YR

- 1.
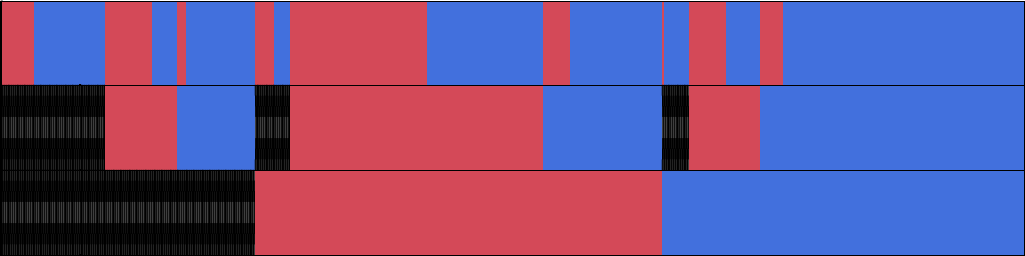
SIFE

Post-I Pre-M 1YR

- 1.
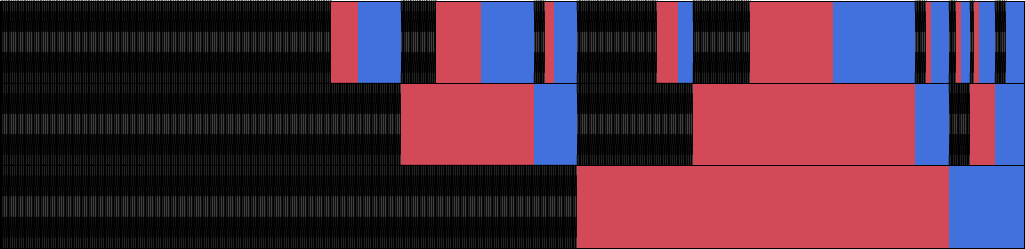
Measurable residual disease Post-I

Pre-M 1YR

Each line (column) represents a separate participant (n=575)
